# Post-exercise cardiac autonomic and cardiovascular responses to heart rate-matched and work rate-matched hypoxic exercise

**DOI:** 10.1007/s00421-021-04678-5

**Published:** 2021-04-03

**Authors:** Alessandro Fornasiero, Andrea Zignoli, Mark Rakobowchuk, Federico Stella, Aldo Savoldelli, Spyros Skafidas, Federico Schena, Barbara Pellegrini, Laurent Mourot

**Affiliations:** 1 CeRiSM, Sport Mountain and Health Research Centre, University of Verona, Via Matteo del Ben, 5/b, 38068 Rovereto, Italy; 2 Department of Neurosciences, Biomedicine and Movement Sciences, University of Verona, Verona, Italy; 3 Department of Industrial Engineering, University of Trento, Trento, Italy; 4 Department of Biological Sciences, Thompson Rivers University Faculty of Science, Kamloops, Canada; 5 EA3920 Prognostic Factors and Regulatory Factors of Cardiac and Vascular Pathologies, Exercise Performance Health Innovation (EPHI) platform, University of Bourgogne Franche-Comté, Besançon, France; 6 National Research Tomsk Polytechnic University, Tomsk, Russia

**Keywords:** Hypoxic exercise, Cardiac baroreflex sensitivity, Heart rate variability, Autonomic nervous system, Post-exercise hypotension, Hypoxia

## Abstract

**Purpose:**

This study investigated the effect of performing hypoxic exercise at the same heart rate (HR) or work rate (WR) as normoxic exercise on post-exercise autonomic and cardiovascular responses.

**Methods:**

Thirteen men performed three interval-type exercise sessions (5 × 5-min; 1-min recovery): normoxic exercise at 80% of the WR at the first ventilatory threshold (N), hypoxic exercise (FiO_2_ = 14.2%) at the same WR as N (H-WR) and hypoxic exercise at the same HR as N (H-HR). Autonomic and cardiovascular assessments were conducted before and after exercise, both at rest and during active squat–stand manoeuvres (SS).

**Results:**

Compared to N, H-WR elicited a higher HR response (≈ 83% vs ≈ 75%HRmax, *p* < 0.001) and H-HR a reduced exercise WR (− 21.1 ± 9.3%, *p* < 0.001). Cardiac parasympathetic indices were reduced 15 min after exercise and recovered within 60 min in N and H-HR, but not after H-WR (*p* < 0.05). H-WR altered cardiac baroreflex sensitivity (cBRS) both at rest and during SS (specifically in the control of blood pressure fall during standing phases) in the first 60 min after the exercise bout (*p* < 0.05). Post-exercise hypotension (PEH) did not occur in H-HR (*p* > 0.05) but lasted longer in H-WR than in N (*p* < 0.05).

**Conclusions:**

Moderate HR-matched hypoxic exercise mimicked post-exercise autonomic responses of normoxic exercise without resulting in significant PEH. This may relate to the reduced WR and the limited associated mechanical/metabolic strain. Conversely, WR-matched hypoxic exercise impacted upon post-exercise autonomic and cardiovascular responses, delaying cardiac autonomic recovery, temporarily decreasing cBRS and evoking prolonged PEH.

**Supplementary Information:**

The online version contains supplementary material available at 10.1007/s00421-021-04678-5.

## Introduction

In the past few years, hypoxic exercise (i.e. exercise combined with hypoxic stress) has been repeatedly highlighted as a promising nonpharmacological therapeutic intervention (Millet et al. 2016; Millet and Girard 2017; Lizamore and Hamlin 2017; Brocherie and Millet 2020). By reducing the mechanical load needed for adequate cardiovascular stimulation, hypoxic exercise represents a suitable option for obese and elderly patients to help meet exercise recommendations (Haufe et al. 2008; Girard et al. 2017; Pramsohler et al. 2017; Hobbins et al. 2017). Hypoxic exercise also has the potential to improve weight loss and cardio-metabolic health in overweight and obese patients (Netzer et al. 2008; Hobbins et al. 2017; Ramos-Campo et al. 2019), further representing a promising approach for insulin resistance and type 2 diabetes prevention and treatment (Mackenzie et al. 2012; De Groote et al. 2018; Mai et al. 2019). Potential applications of hypoxic training in patients with various cardiovascular diseases, including hypertension, have also been highlighted (Wee and Climstein 2015). The present scenario suggests that the number of exercise training interventions including hypoxic exercise is likely to increase in the near future (Millet et al. 2016; Brocherie and Millet 2020). The interest in hypoxic exercise arises from its potential to promote greater physiological and health-related adaptions compared to normoxic exercise (Millet et al. 2016; Girard et al. 2020) in the long-term (i.e. chronic effects), stemming from the markedly different acute physiological responses (i.e. acute effects). At the same absolute work rate (WR), hypoxia-induced arterial chemoreceptor stimulation promotes greater sympathetic activation and withdrawal of parasympathetic activity (Hainsworth et al. 2007; Amann and Kayser 2009; Fisher 2015; Siebenmann et al. 2018), which lead to increased cardiovascular and ventilatory responses (Calbet et al. 2009; Sheel et al. 2010; Fisher 2015; Winkler et al. 2017) to cope with the lower blood oxygen content and to match metabolic demands (Bartsch and Gibbs 2007; Fisher 2015). Compensatory vasodilation of vascular beds facilitating blood and oxygen delivery to the working muscles also occurs (Joyner and Casey 2014; Dinenno 2016). As a result, the same absolute exercise intensity represents a greater physiological challenge in hypoxia (Mazzeo 2008; Fornasiero et al. 2019), which potentially affects post-exercise recovery responses (Luttrell and Halliwill 2015; Romero et al. 2017). An exacerbated increase of exercise-induced physiological stress of hypoxic exercise can result in delayed recovery of autonomic balance (i.e. progressive restoration of normal resting balance between parasympathetic and sympathetic activity) and altered post-exercise cardiovascular responses (Romero et al. 2017; Michael et al. 2017). Delayed parasympathetic recovery has been observed after hypoxic exercise conducted at similar absolute normoxic exercise intensity (Koelwyn et al. 2013; Fornasiero et al. 2018, 2019). In addition, acute post-exercise blood pressure reduction (i.e. post-exercise hypotension, PEH) (Halliwill et al. 2013) may be intensified after hypoxic exercise (Horiuchi et al. 2016, 2018; Saito et al. 2019) leading to an increased risk of orthostatic intolerance and hypotension (Halliwill et al. 2014) in the presence of an affected autonomic blood pressure control (i.e. impaired arterial baroreflex sensitivity). Previous studies investigated the impact of acute hypoxic stimuli on both cardiac and vascular sympathetic baroreflex sensitivity showing contrasting findings (Halliwill and Minson 2002; Roche et al. 2002; Gujic et al. 2007; Querido et al. 2011; Bourdillon et al. 2018; Simpson et al. 2019). However, while it seems that hypoxia may decrease cardiac baroreflex sensitivity (cBRS) (i.e. reduced reflex responsiveness) at rest and during exercise (Roche et al. 2002; Gujic et al. 2007; Bourdillon et al. 2017, 2018), to the best of our knowledge, no study has investigated the impact of hypoxic exercise on post-exercise cBRS restoration. The timeframe for cBRS recovery depends on the physiological exercise intensity (Piepoli et al. 1993; Halliwill et al. 1996; Terziotti et al. 2001; Raczak et al. 2005; Reynolds et al. 2017), which is higher at the same absolute WR in hypoxia. Arguably, both the increased physiological exercise intensity relative to maximal, and hypoxic exercise per se, might negatively affect post-exercise cBRS responses, but this currently remains mere speculation. Indeed, no study has directly investigated post-exercise cBRS responses, comparing normoxic and hypoxic exercises performed at similar relative and absolute exercise intensities. Moreover, studies investigating the impact of hypoxia on cBRS during exercise only compared similar absolute exercise intensities (same WR) (Bourdillon et al. 2017, 2018).

In this regard, a better understanding of autonomic nervous system activity during recovery from hypoxic exercises of various intensities is of paramount importance especially because hypoxic exercise has been recommended amongst vulnerable populations (i.e., elderly and/or overweight/obese people, or suffering from diabetes or hypertension) who often exhibit altered autonomic cardiovascular control, and particularly cardiac baroreflex impairment (Lanfranchi and Somers 2002; Skrapari et al. 2006; Monahan 2007). Also, post-exercise recovery is a critical phase when sudden cardiovascular events are more frequent and often attributable to autonomic disturbances (Luttrell and Halliwill 2015).

Interestingly, the hypoxia-induced increase in cardiovascular and ventilatory stimulation may be mitigated by matching to the exercise heart rate (HR) response (Winkler et al. 2017; Chacaroun et al. 2018; Fornasiero et al. 2019). By adjusting submaximal exercise intensity based on HR during exercise in hypoxia, ventilatory, anaerobic (i.e. blood lactate) and cardiac autonomic responses are similar to normoxic exercise (Zupet et al. 2009; Chacaroun et al. 2018; Fornasiero et al. 2019), and involve reduced mechanical load and aerobic demand (i.e. reduced VO_2_ due to the decreased mechanical load). Similarly, blood pressure is reduced during hypoxic compared to normoxic exercise when HR is matched (Winkler et al. 2017). However, whether HR-matched hypoxic exercise leads to the same post-exercise responses is currently unknown.

Thus, the aim of this study was to examine the effect of performing hypoxic exercise at the same relative (same HR) or same absolute (same WR) normoxic exercise intensity on the post-exercise cardiac autonomic and cardiovascular responses, while recovering in normoxic conditions. We hypothesised that moderate HR-matched hypoxic exercise would be associated with similar post-exercise autonomic and cardiovascular responses when compared to normoxic exercise, whilst WR-matched hypoxic exercise would result in delayed autonomic recovery and post-exercise cardiovascular responses dissimilar to normoxic exercise.

## Methods

### Participants

Thirteen moderately aerobically trained healthy men (age 28 ± 6 years, height 176 ± 6 cm, weight 70.2 ± 5.3 kg, BMI 22.6 ± 1.6 kg/m^2^, $\dot{V}$ O_2max_ 59.3 ± 3.5 mL/min/kg) volunteered for this study. None of the participants had clinical evidence of cardiovascular, metabolic, or musculoskeletal diseases. Before data collection, all participants were properly informed about the experimental protocol and gave their written informed consent. They were instructed to avoid caffeine, alcohol and high-intensity exercise in the 24-h preceding each experimental session. The experimental protocol was approved by the institutional Ethics Committee of the University of Verona (Italy, n°138,232) and performed in accordance with the Declaration of Helsinki.

### Protocol

Each participant visited the laboratory on four occasions (1 preliminary evaluation + 3 experimental sessions) at the same time of the day and completed the experimental protocol within a 4-week period.

A detailed description of the preliminary evaluation, including anthropometric and maximal cardiorespiratory fitness assessment as well as further details of the experimental sessions, has been provided elsewhere (Fornasiero et al. 2019). The preliminary evaluation consisted of a maximal cardiopulmonary exercise test (CPET) under normoxia (10 min at 75 W with increments of 25 W every minute until participants’ volitional exhaustion) to assess maximal exercise work rate (WR) and maximal oxygen uptake ($\dot{V}$ O_2max_). In the experimental protocol, all participants completed three exercise sessions, one in normoxia and two in hypoxia, which were conducted in an environmental chamber under controlled laboratory conditions (21 °C, 50% relative humidity). The hypoxic environment was produced by lowering the fraction of inspired oxygen (FiO_2_) to 14.2%, simulating an altitude of ≈ 3000 m above sea level (a.s.l.), by means of an oxygen dilution system based on the vacuum-pressure swing adsorption principle (B-Cat, Tiel, The Netherlands). Exercise sessions were performed on a cycle ergometer (Excalibur Sport, Lode BV, Groningen, The Netherland) and comprised: 5 min of seated rest for baseline assessment, 5 min of submaximal constant load exercise (warm-up) at 50% of the work rate associated with the first ventilatory threshold (WR@VT1) and five 5-min intervals interspersed by 1 min of passive recovery of either normoxic exercise at 80% WR@VT1(Sugawara et al. 2001) (N), hypoxic exercise with the same absolute WR as during N (H-WR, WR-matched exercise) or hypoxic exercise with the same absolute HR recorded during N (H-HR, HR-matched exercise). A similar design was adopted to obtain key information about cardiorespiratory and cardiac autonomic responses to interval-type hypoxic exercise, which has been presented in a previous study (Fornasiero et al. 2019). Experimental session sequence was partially randomized, since H-HR was always performed after N.

In the environmental chamber, cardiorespiratory measures were collected continuously using an automated, breath-by-breath open-circuit gas analysis system (Quark PFT Ergo, Cosmed Srl, Rome, Italy). Careful calibration of flow sensors and gas analyzers was performed before each measurement according to the manufacturer’s instructions. Pulse oxygen saturation (SpO_2_) was continuously recorded by fingertip pulse oximetry (Nonin Medical, Minneapolis, MN, USA) at a sampling frequency of 1 Hz. To measure blood lactate concentration, a blood sample was collected from the earlobe immediately before the end of each exercise bout (Goodwin et al. 2007). The lactate analyser (Biosen C-line, EKF Diagnostics GmbH, Barleben, Germany) was calibrated according to the manufacturer’s instructions. The individual rating of perceived exertion (RPE) was assessed at the end of each exercise bout using Borg Category Ratio Scale (CR100) (Borg and Borg 2002). Autonomic nervous system and haemodynamic assessments were conducted before entering the environmental chamber and completing the exercise sessions (PRE) and at two different time points during post-exercise recovery in a quiet room under normoxic conditions (23 °C, 50% relative humidity). The setup used in the study was a modified version of the methodology previously described by Mourot et al. (Mourot et al. 2020a) and included cardiac autonomic and haemodynamic assessments at rest (i.e. resting evaluation) and during repeated active squat–stand (SS) manoeuvres (i.e. active evaluation). Resting evaluation consisted of 10 min of seated rest (Terziotti et al. 2001) and was performed at PRE and 15 and 60 min after exercise (POST-15 and POST-60), whilst active evaluation consisted of 5 min of repeated squat–stand manoeuvres with a duty cycle of 10-s squat and 10-s stand (Zhang et al. 2009), which were performed at PRE and 45 and 90 min after exercise cessation (POST-45 and POST-90). Resting and active evaluations were separated by 30 min during which time a non-invasive assessment of vascular function in brachial and femoral arteries was performed. However, these latter data were not included in the present investigation.

During resting phases and SS manoeuvres beat-by-beat blood pressure and R–R intervals were measured continuously using Portapres^®^ device (Finapres Medical System, Amsterdam, The Netherlands) and Polar RS800CX HR monitor (Polar, Kempele, Finland), respectively. BP measurements were taken during resting periods to corroborate Portapres measurements, which were found to be consistent in this sense.

### Data analysis

The R–R intervals were uploaded using Polar Precision Performance Software (Polar, Kempele, Finland) and then exported as.*txt* files. Signal artefacts were filtered out by means of a moderate error correction filter. All the time series of R–R intervals had low noise (identified errors < 5%). HRV analysis was performed using Kubios HRV software (Version 2.1, Biosignal Analysis and Medical Imaging Group, Kuopio, Finland). HRV indices were calculated from the last 5 min of the 10-min resting period. The time-domain HRV indices considered were the square root of the mean squared differences of successive NN intervals (RMSSD) and the standard deviation of normal-to-normal RR intervals (SDNN) (Task Force of the European Society of Cardiology 1996). For frequency-domain HRV indices, low-frequency spectral power (LF, 0.04–0.15 Hz), high-frequency spectral power (HF, 0.15–0.4 Hz), and total spectral power (TP, 0–0.4 Hz) were calculated by fast Fourier transform (FFT) (Task Force of the European Society of Cardiology 1996). Even though the physiological significance of several HRV indices is still disputed (Shaffer and Ginsberg 2017), RMSSD and HF have been extensively used as indices of parasympathetic activity (Task Force of the European Society of Cardiology 1996; Shaffer and Ginsberg 2017).

Inter-beat interval (IBI), beat-to-beat systolic (SAP), diastolic (DAP) and mean (MAP) arterial blood pressure values, as well as other estimated haemodynamic variables (SV, CO and TPR) were extracted using Beatscope Software (TNO-TPD, Biomedical Instrumentation). Haemodynamic data were calculated from the last 5 min of the 10-min period during rest and from the entire 5 min of the squat–stand (SS) manoeuvres. Post-exercise hypotension (PEH) was defined as the absolute difference between SAP at POST and SAP at PRE (PEH = SAP POST–SAP PRE) (Brito et al. 2018).

During repeated SS manoeuvres, maximal (SAP_max_) and minimal (SAP_min_) systolic blood pressure values were extracted from each 20 s cycle (10-s squat–10-s stand) and averaged over the 5-min period (15 cycles).

Beat-by-beat SAP and IBI values were used to assess cBRS as an index of reflex responsiveness.

Custom written Matlab (Mathworks, Natick, MA, ver. 2018a) scripts were used to conduct the following analyses. SAP and IBI data were linearly interpolated and resampled at 2 Hz for spectral and transfer function analysis. Under resting conditions, transfer function analysis (TF) of gain, phase, and coherence between spontaneous oscillations in SAP and IBI was calculated in accordance with the work of (Zhang et al. 2009), i.e. 0.05–0.15 Hz for the low-frequency (LF) range. During SS manoeuvres (performed at 0.05 Hz) TF gain, phase, and coherence were calculated across a specific frequency range (i.e. 0.031–0.078 Hz) (Zhang et al. 2009). cBRS was also assessed with the sequence method (Pinna et al. 2015). The sequence method is based on the identification of at least three consecutive beats (sequence) in which a defined increase (or decrease) in SAP is followed by a defined increase (or decrease) in the IBI. Only sequences with a minimum correlation coefficient of 0.85 were accepted. Positive and negative sequences were averaged to obtain a representative value of cBRS (cBRS_seq_). To better represent blood pressure control in the upward and downward directions, mean gain values of positive (cBRS_Seq_ +) and negative (cBRS_Seq_−) sequences were also computed separately. The two different approaches, i.e. sequence and transfer function methods, were adopted to strengthen our observations and the estimation of cBRS. Both sequence and transfer function methods have been previously adopted to assess spontaneous cBRS under resting condition (Robbe et al. 1987; Pinna et al. 2015). Specifically, sequence method is regarded as the most reliable and the most adopted method to assess spontaneous cBRS at rest (Pinna et al. 2015; Bourdillon et al. 2018), and offers a direct comparison with other studies investigating the impact of hypoxia on cBRS (Subudhi et al. 2014; Bourdillon et al. 2017, 2018). whilst transfer function analysis of SAP and IBI signals in the specific frequency range has been regarded as an appropriated method to assess baroreflex function in response to large perturbations in arterial pressure (e.g. active squat–stand manoeuvres) (Zhang et al. 2009).

### Statistical analysis

Data are presented as means ± standard deviations (SD). Data were tested for normal distribution with the Shapiro–Wilk test. If data were not normally distributed, natural-logarithm transformation (Ln) was applied to obtain a normal distribution and allow parametric statistical comparisons. Autonomic and haemodynamic variables were compared using a two-way ANOVA for repeated measures, with “condition” (N, H-HR, H-WR) and “time” (PRE-POST15-POST60 and PRE-POST45-POST90 for resting and active evaluation, respectively) as within factors. When statistical significance was identified, a Sidak post hoc test was used to further delineate differences between conditions or time. Mean differences (MD) between conditions and time are provided with their 95% confidence interval (CI). The magnitude of the difference (Cohen’s d effect size, ES) was also calculated considering the pooled SDs of pre-exercise (PRE) mean values. Statistical analysis was completed using statistical software (SPSS Inc, Chicago, Illinois, USA). The level of statistical significance was set at *p* < 0.05.

## Results

### Exercise sessions

Detailed results of the exercise sessions are presented elsewhere (Fornasiero et al. 2019).

Work rate used during training sessions was 183 ± 19 W in N and H-WR and 144 ± 18 W in H-HR, with a mean decrement of − 21.1 ± 9.3% (*p* < 0.001). Mean HR during exercise phases was significantly higher in H-WR (154 ± 11 bpm, 83 ± 5% HRmax) compared to N (139 ± 10 bpm; 75 ± 4% HRmax; *p* < 0.001) and H-HR (138 ± 9 bpm; 75 ± 4% HRmax; *p* < 0.001). When compared to *N* (98.2 ± 1.2%) SpO_2_ was reduced to a similar extent both in H-WR (83.2 ± 3.6%, *p* < 0.01) and H-HR (83.6 ± 3.5%, *p* < 0.01). Mean $\dot{V}$ O_2_ was similar in N and H-WR (2.68 ± 0.23 and 2.71 ± 0.23 L/min, respectively, *p* = 0.80) and was reduced in H-HR (2.32 ± 0.24 L/min, *p* < 0.01). Mean $\dot{V}$_E_ was similar in *N* and H-HR (72.1 ± 7.7 and 70.4 ± 9.3 L/min, *p* = 0.81) but was increased in H-WR (88.5 ± 11.4 L/min, *p* < 0.01). Blood lactate concentration was similar in N and H-HR (2.54 ± 1.02 and 2.52 ± 0.87 mmol/L, respectively, *p* = 0.99) and higher during H-WR (4.96 ± 0.91 mmol/L, *p* < 0.01). Compared to N (28.6 ± 9.9) mean RPE was significantly higher in H-WR (36.1 ± 10.5, *p* < 0.018) and lower in H-HR (20.1 ± 7.5; *p* < 0.010).

### Cardiac autonomic modulation responses

**Fig. 1:**
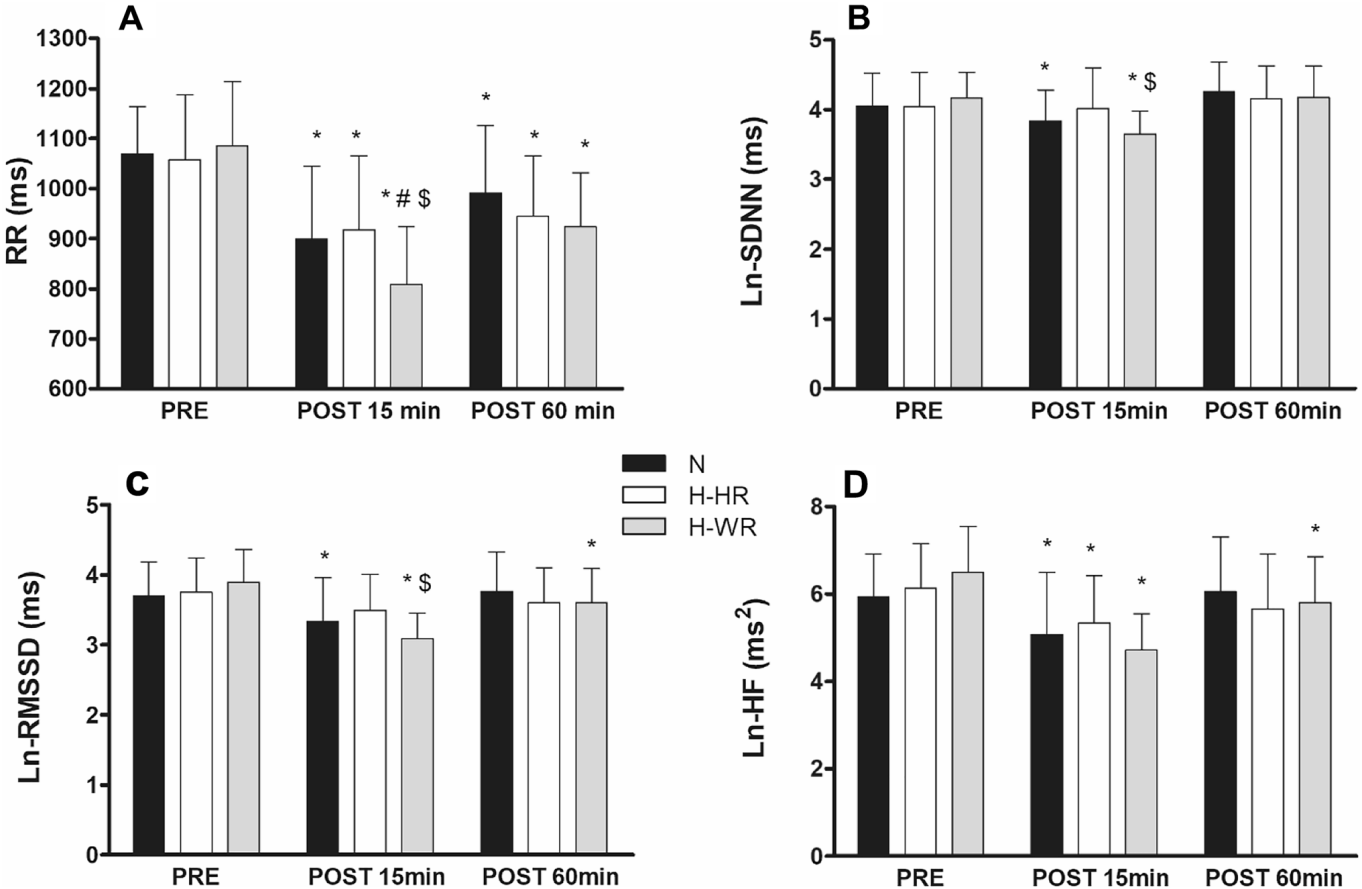
Cardiac autonomic activity before and after the three exercise sessions. Black, white and grey bars represent normoxic exercise (N), heart rate-matched hypoxic exercise (H-HR) and work rate-matched hypoxic exercise (H-WR), respectively. Error bars represent standard deviation of the mean values; #: H-WR ≠ N; $: H-WR ≠ H-HR, *: ≠ PRE; *p* < 0.05; **a**: RR interval; **b**: natural-logarithm transformation of the standard deviation of normal-to-normal R-R intervals (Ln-SDNN); **c**: natural-logarithm transformation of the root mean square of successive differences of R–R intervals (Ln-RMSSD); **d**: natural-logarithm transformation of high-frequency spectral power (Ln-HF);

Cardiac autonomic modulation responses to the three exercise sessions (N, H-HR and H-WR) are presented in Fig. 1. Complete cardiac autonomic modulation responses to the three exercise sessions are provided as electronic supplementary material (ESM 1).

Mean RR interval (RR) exhibited a significant time x condition interaction (*p* < 0.001) with a greater decrease at POST-15 in H-WR compared to *N* (MD − 91 ms, 95% CI − 168 to − 14, *p* = 0.019, ES − 0.77) and H-HR (MD − 110 ms, 95% CI − 186 to − 33, *p* = 0.005, ES − 0.92). Similarly, vagal-related HRV indices (RMSSD and HF) exhibited significant time x condition interactions (*p* < 0.001). Ln-RMSSD was decreased at POST-15 after *N* (MD − 0.36 ms, 95% CI − 0.70 to − 0.01, *p* = 0.041, ES − 0.74) and H-WR (MD − 0.81 ms, 95% CI − 1.12 to − 0.50, *p* < 0.001, ES − 1.69) but not H-HR (*p* = 0.234), and at POST-60 only in response to H-WR protocol (MD − 0.29 ms, 95% CI − 0.55 to − 0.04, *p* = 0.024, ES − 0.67). Ln-HF decreased after all the exercise sessions at POST-15 (*p* < 0.05, ES − 0.84, − 0.78 and − 1.53, for N, H-HR and H-WR, respectively), and only remained reduced after H-WR at POST-60 (MD − 0.68 ms^2^, 95% CI − 1.32 to − 0.04, *p* = 0.036, ES − 0.67). Overall indices of HRV (Ln-SDNN and Ln-TP) also displayed significant time x condition interactions (*p* < 0.05). Specifically, Ln-SDNN decreased at POST-15 in N (MD − 0.21 ms, 95% CI to − 0.42 to 0, *p* = 0.045, ES − 0.48) and H-WR (MD − 0.50 ms, 95% CI − 0.76 to − 0.25, *p* < 0.001, ES − 1.13) but not in H-HR (*p* = 0.998). Similarly, Ln-TP decreased at POST-15 only in H-WR (MD − 0.89 ms^2^, 95% CI − 1.52 to − 0.27, *p* = 0.006, ES − 1.02).

### Blood pressure and baroreflex sensitivity responses at rest

Haemodynamic and cBRS responses to the three exercise sessions (N, H-HR and H-WR) are presented in Table 1.

**Table 1:** Haemodynamic and cardiac baroreflex sensitivity responses during seated rest

	N			H-HR			H-WR		
	PRE	POST 15 min	POST 60 min	PRE	POST 15 min	POST 60 min	PRE	POST 15 min	POST 60 min
SAP (mmHg)	122 ± 12	113 ± 11^a^	121 ± 13	113 ± 11	111 ± 14	116 ± 10^b^	117 ± 14	106 ± 10^a,b^	110 ± 12^a,b,c^
DAP (mmHg)	71 ± 9	69 ± 8	75 ± 10	65 ± 9	67 ± 10	72 ± 8^a^	67 ± 10	66 ± 8	67 ± 9
MAP (mmHg)	90 ± 10	85 ± 9^a^	91 ± 11	83 ± 10	83 ± 11	87 ± 9^a^	85 ± 12	81 ± 9^a^	82 ± 10^b^
CO (L*min^−1^)	5.2 ± 0.8	5.3 ± 1.0	4.8 ± 0.8^a^	5.3 ± 1.0	5.2 ± 1.1	4.9 ± 1.2^a^	5.2 ± 1.1	5.6 ± 1.2	4.9 ± 1.1^a^
SV (mL)	91 ± 12	79 ± 13^a^	79 ± 13^a^	92 ± 12	78 ± 15^a^	75 ± 14^a^	92 ± 15	75 ± 11^a^	75 ± 12^a^
TPR (mmHg s mL^−1^)	1.09 ± 0.24	1.00 ± 0.23	1.19 ± 0.27	0.97 ± 0.26	1.00 ± 0.27	1.15 ± 0.33^a^	1.07 ± 0.32	0.91 ± 0.25^a,c^	1.07 ± 0.33
*Transfer function (TF)*									
Ln-cBRS_TF_-gain (ms*mmHg^−1^)	2.35 ± 0.35	2.25 ± 0.55	2.37 ± 0.38	2.38 ± 0.34	2.37 ± 0.52	2.43 ± 0.38	2.39 ± 0.36	2.11 ± 0.29^a^	2.32 ± 0.34
cBRS_TF_-phase (rads)	− 0.43 ± 0.20	− 0.51 ± 0.23	− 0.50 ± 0.20	− 0.49 ± 0.24	− 0.58 ± 0.27	− 0.55 ± 0.26	− 0.37 ± 0.14	− 0.56 ± 0.22	− 0.48 ± 0.25
cBRS_TF_-coherence	0.66 ± 0.12	0.67 ± 0.08	0.58 ± 0.17	0.65 ± 0.13	0.62 ± 0.17	0.55 ± 0.19	0.66 ± 0.13	0.67 ± 0.11	0.62 ± 0.13
*Sequence method*									
*n* seq +	13 ± 4	19 ± 6^a^	16 ± 4	12 ± 3	16 ± 4^a^	16 ± 4	15 ± 6	21 ± 7^a^	17 ± 3
*n* seq_−_	14 ± 3	18 ± 6^a^	16 ± 4	14 ± 4	17 ± 4^a^	16 ± 5	16 ± 7	22 ± 10^a^	17 ± 3
Ln-cBRS_seq_ + (ms*mmHg^−1^)	2.32 ± 0.52	2.28 ± 0.49	2.54 ± 0.54	2.59 ± 0.66	2.49 ± 0.60	2.46 ± 0.49	2.50 ± 0.52	2.11 ± 0.30^a,c^	2.44 ± 0.51
Ln-cBRS_seq−_ (ms*mmHg^−1^)	2.33 ± 0.60	2.22 ± 0.56	2.52 ± 0.52	2.32 ± 0.56	2.51 ± 0.61	2.46 ± 0.57	2.40 ± 0.46	2.14 ± 0.34^a^	2.38 ± 0.44
Ln-cBRS_seq_ (ms*mmHg^−1^)	2.34 ± 0.54	2.26 ± 0.52	2.54 ± 0.50	2.50 ± 0.57	2.50 ± 0.59	2.48 ± 0.51	2.45 ± 0.47	2.13 ± 0.32^a,c^	2.41 ± 0.47

Values are means ± SD. Transfer function gain, phase, and coherence values were estimated in the low-frequency range (LF) from 0.05 to 0.15 Hz

*SAP* systolic arterial pressure* , DBP* diastolic arterial pressure, *MAP* mean arterial pressure, *CO* cardiac output, *SV* stroke volume, *TPR* total peripheral resistance, *cBRS* cardiac baroreflex sensitivity, *TF* transfer function, *Seq* sequence method, + : up sequences, −: down sequences

*p* < 0.05

^a^ ≠ PRE; ^b^: ≠ N; ^c^ ≠ H-HR

**Fig. 2:**
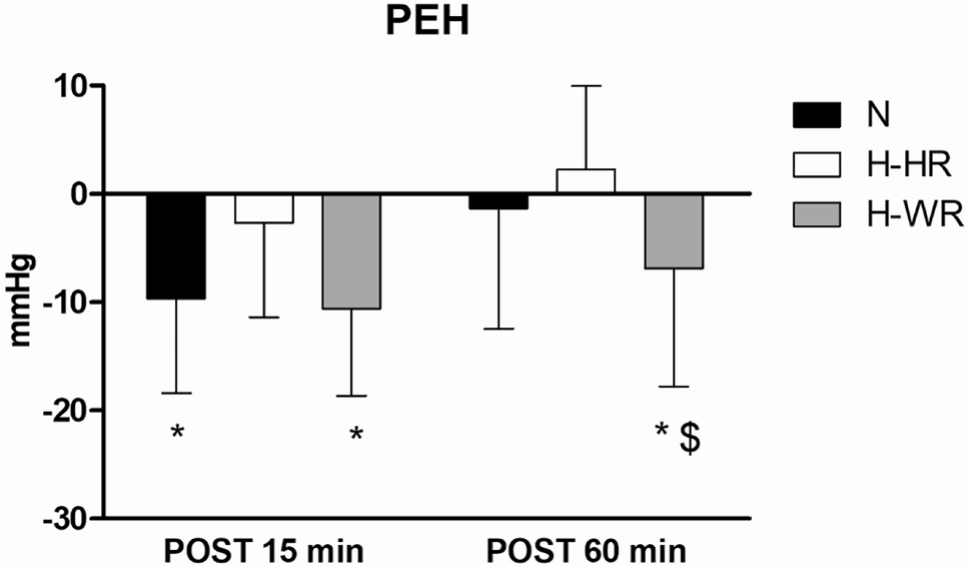
Post-exercise hypotension (PEH) responses evoked by the three exercise sessions. Black, white and grey bars represent normoxic exercise (N), heart rate-matched hypoxic exercise (H-HR) and work rate-matched hypoxic exercise (H-WR), respectively. Error bars represent standard deviation of the mean values. *: ≠ pre-exercise value; # ≠ N, $: ≠ H-HR; *p* < 0.05. PEH is defined as the absolute difference between systolic arterial pressure (SAP) at POST and SAP at PRE (PEH = SAP POST–SAP PRE)

SAP exhibited a significant time x condition interaction (*p* = 0.020). Specifically, compared to PRE (Fig. 2), SAP decreased at POST-15 in N (MD − 10 mmHg, 95% CI − 16 to − 3, *p* = 0.005, ES − 0.77) and H-WR (MD − 11 mmHg, 95% CI − 17 to − 4, *p* = 0.001, ES − 0.85) and not in H-HR (*p* = 0.807), and at POST-60 only in H-WR (MD − 7 mmHg, 95% CI − 17 to 0, *p* = 0.043, ES − 0.55).

Similar to SAP, DAP displayed a significant time x condition interaction (*p* = 0.014). Compared to PRE, DAP was significantly increased at POST-60 in H-HR (MD 7 mmHg, 95% CI 3–11, *p* = 0.002, ES 0.72). Moreover, MAP exhibited a significant time x condition interaction (*p* = 0.026) with a decrease at POST-15 in N (MD -5 mmHg, 95% CI − 10 to − 1, *p* = 0.024, ES − 0.48) and H-WR (MD − 4 mmHg, 95% CI − 9 to 0, *p* = 0.037, ES − 0.41), not in H-HR (*p* = 0.955), and increased at POST-60 in H-HR (MD 5 mmHg, 95% CI 1 to 9, *p* = 0.017, ES 0.45) compared to PRE. At POST-60 MAP was significantly lower in H-WR compared to N (MD − 9 mmHg, 95% CI − 18 to 0, *p* = 0.044, ES − 0.83).

CO significantly decreased at POST-60 compared to PRE (main effect for time *p *= 0.002) and SV was significantly reduced at POST-15 and POST-60 compared to PRE (main effects for time all *p* < 0.001). TPR exhibited a significant time x condition interaction (*p* = 0.035) with decreases at POST-15 in H-WR (MD − 0.16 mmHg s mL^−1^, 95% CI − 0.28 to − 0.05, *p* = 0.009, ES − 0.56) and increases at POST-60 in H-HR (MD 0.18 mmHg s mL^−1^, 95% CI 0.06 to 0.29, *p* = 0.003, ES 0.61) compared to PRE.

cBRS assessed by means of transfer function analysis (Ln-cBRS_TF_) displayed a significant time x condition interaction (*p* = 0.018) with a decreased gain at POST-15 only in H-WR (MD − 0.28 ms*mmHg^−1^, 95% CI − 0.47 to − 0.09, *p* = 0.004, ES − 0.79). Similarly, Ln-cBRS_seq_ significantly decreased (time x condition interaction *p* = 0.020) at POST-15 in H-WR (MD − 0.32 ms*mmHg^−1^, 95% CI − 0.60 to − 0.04, *p* = 0.027, ES − 0.61) and not in H-HR (*p* = 0.998) nor N (*p* = 0.863). At POST-15 Ln-cBRS_seq_ was significantly different in H-WR compared to H-HR (MD − 0.37 ms*mmHg^−1^, 95% CI − 0.76 to − 0.02, *p* = 0.045, ES − 0.71). A significant time x condition interaction was also found for Ln-cBRS_seq_ + (*p* = 0.027) and Ln-cBRS_seq−_ (*p* = 0.036), which decreased at POST-15 min only in H-WR (MD − 0.38 ms*mmHg^−1^, 95% CI − 0.70 to − 0.06, *p* = 0.018, ES − 0.68 and MD − 0.24 ms*mmHg^−1^, 95% CI − 0.46 to − 0.01, *p* = 0.038, ES − 0.43, for Ln-cBRS_seq_ + and Ln-cBRS_seq−_, respectively). The number of positive (*n* +) and negative (*n*−) sequences increased at POST-15 after all exercise sessions (time effect, *p* < 0.05).

### Blood pressure and baroreflex sensitivity responses to repeated squat–stand test manoeuvres

Blood pressure and cBRS responses to repeated squat–stand test manoeuvres in the three exercise sessions (N, H-HR and H-WR) are presented in Table 2.

**Table 2:** Haemodynamic and cardiac baroreflex sensitivity responses during repeated squat–stand manoeuvres

	N			H-HR			H-WR		
	PRE	POST 45 min	POST 90 min	PRE	POST 45 min	POST 90 min	PRE	POST 45 min	POST 90 min
SAP	134 ± 14	125 ± 11^a^	132 ± 11	128 ± 13	124 ± 14	131 ± 13	129 ± 12	116 ± 14^a,b;c^	123 ± 12^a,b,c^
DAP	75 ± 9	73 ± 9	75 ± 10	70 ± 9	70 ± 8	75 ± 9^a^	71 ± 9	67 ± 9^a,b;c^	72 ± 8^c^
MAP	97 ± 10	92 ± 10^a^	96 ± 11	91 ± 10	89 ± 10	96 ± 10^a^	92 ± 10	84 ± 11^a,b;c^	91 ± 9^c^
HR	73 ± 7	84 ± 10^a^	82 ± 10^a^	77 ± 8	84 ± 10^a^	82 ± 9^a^	75 ± 10	89 ± 13^a,b;c^	85 ± 13^a^
SAP_max−_squat	165 ± 17	162 ± 14	168 ± 12	164 ± 14	162 ± 13	167 ± 15	163 ± 13	160 ± 12	159 ± 18
SAP_min−_stand	111 ± 16	99 ± 12^a^	107 ± 10	104 ± 16	97 ± 17	103 ± 15	103 ± 15	84 ± 17^a,b;c^	98 ± 18
*Transfer function*									
Ln-cBRS_TF_-gain	2.03 ± 0.33	2.03 ± 0.34	2.00 ± 0.30	1.93 ± 0.29	1.90 ± 0.28	1.93 ± 0.22	2.03 ± 0.29	1.91 ± 0.19	2.07 ± 0.25
cBRS_TF_-phase	− 0.51 ± 0.17	− 0.49 ± 0.22	− 0.47 ± 0.26	− 0.50 ± 0.14	− 0.68 ± 0.29	− 0.73 ± 0.24	− 0.67 ± 0.34	− 0.76 ± 0.28	− 0.66 ± 0.33
cBRS_TF_-coherence	0.66 ± 0.09	0.69 ± 0.08	0.66 ± 0.08	0.68 ± 0.09	0.65 ± 0.07	0.65 ± 0.08	0.71 ± 0.07	0.68 ± 0.08	0.69 ± 0.07
*Sequence method*									
Ln-cBRS_seq_ +	1.96 ± 0.25	1.95 ± 0.24	1.98 ± 0.24	1.91 ± 0.21	1.87 ± 0.17	1.94 ± 0.17	1.94 ± 0.29	1.89 ± 0.17	1.99 ± 0.18
Ln-cBRS_seq−_	1.75 ± 0.30	1.65 ± 0.29	1.71 ± 0.33	1.65 ± 0.28	1.58 ± 0.26	1.67 ± 0.19	1.69 ± 0.35	1.42 ± 0.24^a,b^	1.67 ± 0.33
Ln-cBRS_seq_	1.86 ± 0.27	1.81 ± 0.26	1.85 ± 0.28	1.79 ± 0.23	1.74 ± 0.20	1.81 ± 0.16	1.82 ± 0.31	1.68 ± 0.17	1.85 ± 0.24

Values are means ± SD. Transfer function gain, phase, and coherence values were estimated in the squat–stand manoeuvres frequency range from 0.031 to 0.078 Hz

*SAP* systolic arterial pressure, *DAP* diastolic arterial pressure, *MAP* mean arterial pressure, *HR* heart rate, *cBRS* cardiac baroreflex sensitivity, *TF* transfer function, *Seq*: sequence method,  + : up sequences, −: down sequences

*p* < 0.05

^a^: ≠ PRE; ^b^: ≠ *N*; ^c^ ≠ H-HR

During SS, mean SAP significantly decreased (time x condition interaction *p* = 0.018) at POST− 45 in N (MD − 9 mmHg, 95% CI − 16 to − 2, *p* = 0.010, ES − 0.68) and H-WR (MD − 13 mmHg, 95% CI − 18 to − 8, *p* < 0.001, ES − 0.98) but not in H-HR (*p* = 0.307), and to a greater extent in H-WR compared to N (MD − 9 mmHg, 95% CI − 15 to − 3, *p* = 0.003, ES − 0.70). SAP was still decreased at POST-90 only in H-WR (MD − 6 mmHg, 95% CI − 10 to − 1, *p* = 0.018, ES − 0.43) with reduced values compared to N and H-HR (MD -8 mmHg, 95% CI − 17 to 0, *p* = 0.042, ES − 0.64 and MD − 8 mmHg, 95% CI − 15 to − 1, *p* = 0.027, ES − 0.59, compared to N and H-HR, respectively). SAP_min_ significantly decreased (time x condition interaction *p* = 0.011) at POST-45 in N (MD − 12 mmHg, 95% CI − 20 to − 5, *p* = 0.002, ES − 0.79) and H-WR (MD − 20 mmHg, 95% CI − 28 to − 11, *p* < 0.001, ES − 1.25) but not in H-HR (MD -7 mmHg, 95% CI − 15 to 1, *p* = 0.080, ES − 0.44), with reduced values in H-WR compared to N (MD − 15 mmHg, 95% CI − 22 to − 8, *p* < 0.001, ES − 0.96).

**Fig. 3:**
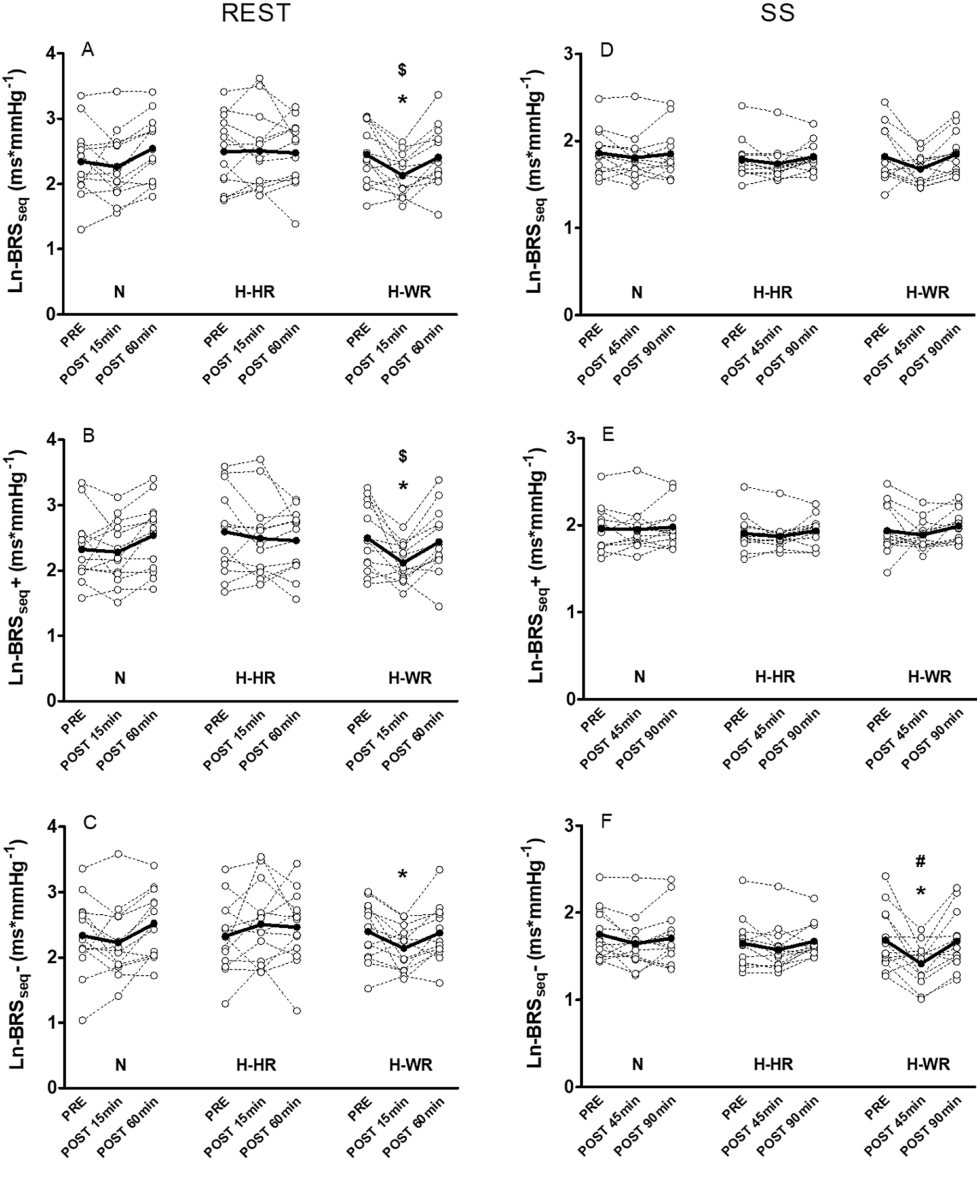
Cardiac baroreflex sensitivity (cBRS) responses (sequence method) before and after the three exercise sessions during seated rest (REST) and active squat–stand manoeuvres (SS). Individual (white circles) and mean (black circles and lines) responses are shown. *: ≠ PRE; #: ≠ N; $: ≠ H-HR; *p* < 0.05. **a**: Mean gain of up- and down-cBRS sequences during seated rest; **b**: mean gain of up-cBRS sequences during seated rest; **c**: mean gain of down-cBRS sequences during seated rest; **d**: mean gain of up- and down-cBRS sequences during squat–stand manoeuvres; **e**: mean gain of up-cBRS sequences during squat–stand manoeuvres; **f**: mean gain of down-cBRS sequences during squat–stand manoeuvres

Compared to PRE, DAP significantly decreased (time x condition interaction (*p* = 0.013) at POST-45 in H-WR (MD − 4 mmHg, 95% CI − 8 to 0, *p* = 0.035, ES − 0.44) and increased at POST-90 in H-HR (MD 5 mmHg, 95% CI 1 to 9, *p* = 0.017, ES 0.56). Similarly, compared to PRE, MAP significantly decreased (time x condition interaction (*p* = 0.038) at POST-45 in N (MD − 5 mmHg, 95% CI − 10 to 0, *p* = 0.031, ES − 0.51) and H-WR (MD − 8 mmHg, 95% CI − 12 to − 4, *p* = 0.001, ES − 0.75), not in H-HR (*p* = 0.638), and increased at POST-90 only in H-HR (MD 4 mmHg, 95% CI 1 to 8, *p* = 0.042, ES 0.52). MAP was significantly lower in H-WR compared to N (MD − 7 mmHg, 95% CI − 12 to − 3, *p* = 0.003, ES − 0.72) and H-HR (MD − 5 mmHg, 95% CI − 7 to − 3, *p* < 0.001, ES − 0.50) at POST-45 and compared to H-HR at POST-90 (MD –  5 mmHg, 95% CI − 9 to − 1, *p* = 0.018, ES − 0.49). No significant differences were apparent in cBRS gain, phase and coherence of transfer function analysis during SS manoeuvres across the exercise sessions (*p* > 0.05). Conversely, cBRS_seq−_ exhibited a significant time x condition interaction (*p* = 0.032), which decreased at POST-45 only with H-WR (MD − 0.27 ms*mmHg^−1^, 95% CI − 0.51 to − 0.02, *p* = 0.030, ES − 0.86) (Fig. 3).

## Discussion

The purpose of this study was to investigate the effects of performing hypoxic exercise (interval-type exercise, 5 × 5-min exercise with 1-min rest, FiO_2_ = 14.2%) at the same relative (HR-matched) and same absolute (WR-matched) normoxic exercise intensity on post-exercise cardiac autonomic and cardiovascular responses. The key findings were that: (1) post-exercise responses from moderate HR-matched hypoxic exercise (~ 75% HRmax) were similar to responses during normoxic exercise. cBRS remained unchanged, while cardiac parasympathetic indices decreased and recovered within 60 min after exercise in the two exercise modalities. However, contrary to normoxic exercise, HR-matched hypoxic exercise, associated with a reduced mechanical WR (− 21%), did not result in post-exercise hypotension (PEH). (2) WR-matched hypoxic exercise, associated with greater physiological stimulation (~ 83% HRmax, + 15 bpm), delayed cardiac autonomic recovery (parasympathetic indices still decreased 60 min after exercise), decreased cBRS at rest and during repeated squat–stand (SS) manoeuvres (recovered within 45 min after exercise) and evoked longer PEH (still present 60 min after exercise). Additionally, during this dynamic task, wider fluctuations in blood pressure, greater post-exercise hypotension and reduction in cBRS were also apparent, specifically during standing phases (i.e. reduction in control of blood pressure fall).

In an intensity-dependent fashion during aerobic exercise, the cardiovascular responses required to meet metabolic needs (Nobrega et al. 2014; Fisher et al. 2015) are mediated by progressive cardiac parasympathetic withdrawal and sympathetic activation (White and Raven 2014). Heart rate (HR), stroke volume (SV), cardiac output (CO) and arterial pressure (AP) elevation, combined with vasoconstriction of viscera and non-active muscles, ensure adequate perfusion of exercising muscles, where, conversely, vasodilation occurs (i.e. functional sympatholysis) (Nobrega et al. 2014; Fisher et al. 2015). After exercise, a progressive decrease in HR and CO occurs due to cardiac parasympathetic reactivation and sympathetic withdrawal (Michael et al. 2017). Alongside these alterations, increases in vascular conductance due to a combination of centrally mediated (i.e. decrease in sympathetic outflow) and persistent local vasodilator mechanisms reduce AP below pre-exercise levels, i.e. to post-exercise hypotension (PEH) (Halliwill et al. 2013; Luttrell and Halliwill 2015).

It has been repeatedly reported that HR (and HRV) and blood pressure monitoring in the post-exercise period provides important non-invasive indices of autonomic function (Luttrell and Halliwill 2015; Romero et al. 2017; Michael et al. 2017), revealing the impact of the previous exercise stimulus on autonomic disturbance and subsequent recovery. These indices have been further shown to provide meaningful information about the added autonomic disturbance induced by exercising with environmental stressors, such as cold (Sanchez-Gonzalez and Figueroa 2013), heat (Pecanha et al. 2017) and hypoxia (Fornasiero et al. 2018).

For the same submaximal work rate in hypoxia greater sympathetic activation and parasympathetic withdrawal is induced by arterial chemoreceptor stimulation (Amann and Kayser 2009; Nobrega et al. 2014; Siebenmann et al. 2018) with further increases in HR, SV, CO, and AP (Calbet et al. 2009; Fisher 2015; Winkler et al. 2017), and greater respiratory involvement (Sheel et al. 2010; Fornasiero et al. 2019). These heightened responses, coupled with the so-called ‘compensatory vasodilation’ (Joyner and Casey 2014; Dinenno 2016), ensure adequate perfusion of active muscles during hypoxic exercise, but represent additional cardiovascular and autonomic stresses and may translate into delayed post-exercise autonomic and cardiovascular responses (Koelwyn et al. 2013; Horiuchi et al. 2016, 2018; Fornasiero et al. 2018, 2019; Saito et al. 2019). An exacerbated increase of exercise-induced physiological stress has been shown to delay post-exercise cardiac autonomic recovery after high-intensity hypoxic exercise and after exercises conducted at similar absolute normoxic exercise intensities in hypoxia (Koelwyn et al. 2013; Fornasiero et al. 2018, 2019). Nevertheless, we recently observed (Fornasiero et al. 2019) that when cardiorespiratory stimulation is matched (i.e. similar cardiac, ventilatory and blood lactate responses), cardiac autonomic responses (exercise and recovery HRV responses during interval-type exercise) are not different in hypoxia compared to normoxia. These previous findings underlined that moderate HR-matched hypoxic exercise may not result in greater post-exercise autonomic disturbance (Fornasiero et al. 2019).

Accordingly, and in line with our hypotheses, we observed a delayed post-exercise cardiac autonomic recovery after H-WR, but not after H-HR. Indeed, a larger decrease in resting RR interval compared to N, without complete parasympathetic recovery (i.e. Ln-RMSSD and Ln-HF) 60 min after exercise were observed in H-WR. This is in agreement with previous investigations suggesting that cardiac autonomic recovery is profoundly influenced by exercise intensity, with higher exercise intensities (above VT) delaying cardiac autonomic recovery (Terziotti et al. 2001; Seiler et al. 2007; Michael et al. 2016). Interestingly, although WR-matched exercise exhibited differences between hypoxic and normoxic conditions, H-HR was associated with neither delayed post-exercise cardiac autonomic recovery nor affected cBRS responses. Mean RR interval and vagal-related HRV indices (Ln-HF) decreased to a similar extent in H-HR and N 15 min after exercise. Moreover, vagal-related HRV indices were completely recovered 60 min after exercise both after H-HR and N, suggesting similar parasympathetic recovery for the two conditions.

On the other hand, the delayed recovery of parasympathetic indices in H-WR was associated to decreased cBRS at rest, and during the dynamic stimulation induced by SS manoeuvres (i.e. wider fluctuations in blood pressure), mirroring impaired autonomic control of blood pressure (i.e. reduced reflex responsiveness). Gains of cBRS_seq_ + , cBRS_seq−_ and total cBRS sequences, as well as cBRS gain calculated using the transfer function approach (cBRS_TF_), were indeed altered at rest in response to H-WR trial. Conversely, during repeated SS manoeuvres, only gain of negative sequences (Ln-cBRS_seq−_) was decreased, indicating a specific decrease in the control of decreasing blood pressure (i.e. blood pressure fall during standing phases).

cBRS is important in maintaining AP, and often post-exercise alterations depend upon the previous exercise intensity (Piepoli et al. 1993; Halliwill et al. 1996; Terziotti et al. 2001; Raczak et al. 2005; Reynolds et al. 2017). For instance, 30 min of exercise at 65% of HRmax leads to cBRS improvement (Raczak et al. 2005). Conversely, high-intensity (> 85% HRmax) and maximal aerobic exercises often decrease cBRS with recovery by 60 min after exercise cessation (Somers et al. 1985; Piepoli et al. 1993; Terziotti et al. 2001; Reynolds et al. 2017). Within the moderate intensity range used during N (~ 75% HRmax), post-exercise cBRS responses have been shown to be more variable (Halliwill et al. 2013). For example, cBRS improvements may occur after moderate exercise at the same intensity as in our study (Halliwill et al. 1996) but with a longer duration (60 min vs 5 × 5 min here). Hence, our results are in line with the documented cBRS responses following normoxic exercise of moderate intensity.

Overall, and for the first time, these findings attest similar post-exercise cardiac autonomic recovery and similar autonomic blood pressure control after hypoxic and normoxic exercises conducted at a similar moderate HR (~ 75% of HRmax). Our findings suggest that the prescription of moderate exercise intensities based on HR in hypoxia can help control the increased exercise-induced physiological stress of hypoxic exercise and limit its impact on post-exercise cardiac autonomic recovery. These results highlight the usefulness of HR-matched hypoxic exercise as a means to control the additional stress imposed by hypoxia on autonomic function, which may result particularly relevant for clinical populations (Millet et al. 2016; Brocherie and Millet 2020). PEH has been documented after exercises of different type, intensity and duration (Halliwill et al. 2013; Romero et al. 2017), and was an expected phenomenon in response to the protocol adopted in our study (≈ 30 min of exercise at moderate intensity). Indeed, PEH [calculated from systolic arterial pressure change (Brito et al. 2018)] was present (-9 mmHg) at rest 15 min after exercise, and during SS manoeuvres 45 min after exercise for N condition.

One of the main findings of present study is that no PEH was observed after H-HR, whilst longer PEH manifestation was evident for H-WR trial. After H-HR the lack of PEH was accompanied by an increase in DAP and MAP compared to pre-exercise levels 60 min after exercise. These peculiar responses might be attributable to the prolonged sitting posture of our participants due to our study design (Halliwill et al. 2013; Brito et al. 2018), which might partially counteract the hypotensive effect of exercise during seated recovery (Halliwill et al. 2013). This would further explain the decreased SV and CO observed during seated recovery 60 min after exercise cessation (Halliwill et al. 2013).

In the literature, the impact of hypoxic exercise on PEH has been poorly studied. Previous studies reported both amplified (Horiuchi et al. 2016, 2018; Saito et al. 2019) and similar PEH (Kleinnibbelink et al. 2020) after hypoxic exercise, but it is worth noting that these previous studies differ from the present investigation either in the recovery modality (hypoxia) (Horiuchi et al. 2016; Saito et al. 2019; Kleinnibbelink et al. 2020) or exercise type (i.e. resistance exercise) (Horiuchi et al. 2018). Most current suggestions to use hypoxic exercise as a training modality (Millet et al. 2016; Millet and Girard 2017; Lizamore and Hamlin 2017; Brocherie and Millet 2020) suggest performing the recovery under normoxic conditions, like in the present study. To the best of our knowledge, no studies have investigated PEH following hypoxic exercise (aerobic) with normoxic recovery, making any comparison with the present investigation difficult. For example, Saito et al. (2019) found greater and longer PEH effects after a maximal exercise (to exhaustion) under hypobaric hypoxia (~ 2200 m) compared to maximal exercise in normoxia and submaximal exercise in normoxia matched for the total volume of hypoxic exercise (~ 10 min). In that study, reductions in SAP and MAP were still present 60 min after the hypoxic trial only. In addition, Horiuchi et al. (2016) found a more pronounced decrease in MAP (-3 mmHg average during 60 min recovery, − 5 mmHg 60 min post-exercise) after 2 h of moderate intensity exercise (4 × 30 min exercise with 15-min recovery) at 50% of altitude-adjusted $\dot{V}$ O_2 max_ in hypoxia (FiO_2_ = 14.1%) compared to normoxia. Both the higher intensity (Saito et al. 2019), the longer duration of exercise (Horiuchi et al. 2016), and the recovery modality (hypoxia) might explain the differences with the results from the present study.

According to our results, both the increased stimulation (i.e. physiological) of H-WR and the reduced stimulation (i.e., mechanical/metabolic,) of H-HR played a role on the PEH responses evoked. In this study, the greater physiological stimulation of H-WR translated into longer PEH, which seems to be in line with previous observations (Horiuchi et al. 2016; Saito et al. 2019), but with the confounding factor of a higher exercise intensity (Halliwill et al. 2013). Indeed, in this study, the greater physiological exercise intensity of H-WR resulted in post-exercise autonomic alterations, that together with a probable prolonged vasodilation, as indirectly supported by reduced TPR, might contribute to the longer PEH observed. On the other hand, the reduced work rate (and total work) of H-HR translated into reduced PEH. This occurrence may be relevant for both short- and long-term blood pressure reduction. Indeed, PEH is by itself a beneficial short-term reduction of blood pressure of clinical relevance (Brito et al. 2018), which has been shown to predict blood pressure reduction to chronic exercise training in prehypertensive (Liu et al. 2012) and healthy (Hecksteden et al. 2013; Brito et al. 2018) individuals. According to our findings, a longer exercise duration, matching the total work done (Jones et al. 2007), may be required to evoke PEH after hypoxic exercise performed at a similar HR of normoxic exercise. This may be particularly true for exercise sessions of short duration (as the present investigation, ~ 30 min) and of moderate intensity. However, our results derive from a group of healthy normotensive individuals and, therefore, further research is needed to address this issue in prehypertensive and hypertensive individuals, where PEH magnitude might differ (Brito et al. 2018). Future studies should, therefore, focus on the investigation of the interplay between exercise intensity (i.e. physiological and mechanical) and other exercise characteristics in inducing (a desired level of) PEH in response to different protocols of hypoxic exercise. This would help anticipate the post-exercise autonomic and cardiovascular outcomes induced by hypoxic exercise and increase its applicability in a wide range of contexts and clinical populations.

## Limitations

Different exercises, in terms of type, intensity and duration, different degrees of hypoxia, as well as different body positions assumed during recovery could result in different autonomic and cardiovascular responses (Halliwill et al. 2013; Michael et al. 2017). Accordingly, the results obtained in this study may be limited to the specific exercise and the post-exercise recovery modality (seated recovery in normoxia) performed by the participants. In addition, as we did not directly evaluate muscle sympathetic nerve activity (MSNA) or blood catecholamines responses, we cannot properly evaluate sympathetic influence on the observed responses. The specific contribution of the peripheral circulation and vasodilation should also be studied.

Regarding HRV measurements, it is worth noting that we did neither control nor determine breathing frequency during the pre/post-exercise assessments. We know that the respiratory involvement was greater in H-WR, and similar in H-HR, compared to N, but it is reasonable to think that the respiratory pattern was not significantly different during the post-exercise assessment (i.e. 15–60 min post-exercise). Indeed, we found that HF_peak_ (Hz), estimated by means of FFT analysis, did not change among the investigated conditions (please see ESM1). We also know that altered respiratory patterns mostly influence frequency-domain HRV indices (e.g. HF power) and not time-domain indices (e.g. RMSSD) (Penttilä et al. 2001). Therefore, we believe that there is enough evidence to support the idea of a similar and slower (compared to N) parasympathetic recovery in H-HR and H-WR, respectively.

The participants in this study were healthy active men and, therefore, these findings may not be directly generalizable to sedentary, clinical, or elderly populations. Similarly, due to existing gender-related differences in cardiac autonomic and cardiovascular responses to exercise (Mourot et al. 2020b) and hypoxia (Richalet et al. 2020) between male and female subjects, the results of our study might be hardly generalizable to women.

## Conclusions

Moderate HR-matched hypoxic exercise (~ 75% HRmax, FiO_2_ = 14.2%) did not affect cBRS and did not blunt cardiac autonomic recovery during the early post-exercise recovery phase. However, post-exercise hypotension was absent and may relate to the reduced WR (− 21%) and the limited associated mechanical/metabolic strain. Conversely, WR-matched hypoxic exercise, resulting in greater physiological stimulation (~ 83% HRmax), delayed cardiac autonomic recovery, temporarily decreased cBRS and evoked longer post-exercise hypotension. Post-exercise autonomic and cardiovascular responses to different HR-matched and WR-matched hypoxic exercises warrant further investigation, especially in clinical populations.

## Supplementary Information

Below is the link to the electronic supplementary material.

Supplementary file1 (DOCX 20 KB)

## Abbreviations

ANOVA: Analysis of variance
ANS: Autonomic nervous system
cBRS: Cardiac baroreflex sensitivity
HR: Heart rate
HRV: Heart rate variability
Ln: Natural-logarithm transformation
PEH: Post-exercise hypotension
RMSSD: Root mean square of successive differences of R–R intervals
RPE: Rating of perceived exertion
SS: Squat–stand
