# Supplementary material for: Post-exercise cardiac autonomic and cardiovascular responses to heart rate-matched and work rate-matched hypoxic exercise
Source: Eur J Appl Physiol. 2021 Apr 3;121(7):2061–76. doi: 10.1007/s00421-021-04678-5 (PMC8192382; doi:10.1007/s00421-021-04678-5)
Supplement: Supplementary file 1 — Supplementary file1 (DOCX 20 KB) [file 421_2021_4678_MOESM1_ESM.docx]

| **ESM 1. Cardiac autonomic modulation responses before and after the three exercise sessions during seated rest** | | | | | | | | | | | | | | | | | | | | | | | | | | | | | | | | | | | | |
| --- | --- | --- | --- | --- | --- | --- | --- | --- | --- | --- | --- | --- | --- | --- | --- | --- | --- | --- | --- | --- | --- | --- | --- | --- | --- | --- | --- | --- | --- | --- | --- | --- | --- | --- | --- | --- |
|  | N | | | | | | | | | | | H-HR | | | | | | | | | | | | H-WR | | | | | | | | | | | | |
|  | PRE | | | POST 15 min | | | | POST 60 min | | | | PRE | | | POST 15 min | | | | POST 60 min | | | | | PRE | | | | POST 15 min | | | | | POST 60 min | | | |
| RR (ms) | 1069 | ± | 94 | 899 | ± | 145 | * | 991 | ± | 134 | * | 1058 | ± | 130 | 918 | ± | 147 | * | 944 | ± | 121 | * | 1085 | | ± | 128 | 808 | | ± | 115 | *,#,$ | 924 | | ± | 108 | * |
| Ln-SDNN (ms) | 4.05 | ± | 0.47 | 3.84 | ± | 0.44 | * | 4.26 | ± | 0.42 |  | 4.04 | ± | 0.49 | 4.02 | ± | 0.58 |  | 4.15 | ± | 0.47 |  | 4.16 | | ± | 0.37 | 3.65 | | ± | 0.33 | *,$ | 4.17 | | ± | 0.45 |  |
| Ln-RMSSD (ms) | 3.69 | ± | 0.49 | 3.33 | ± | 0.62 | * | 3.76 | ± | 0.56 |  | 3.75 | ± | 0.49 | 3.49 | ± | 0.51 |  | 3.61 | ± | 0.49 |  | 3.89 | | ± | 0.46 | 3.08 | | ± | 0.37 | *,$ | 3.60 | | ± | 0.49 | * |
| HF_peak_ (Hz) | 0.25 | ± | 0.07 | 0.22 | ± | 0.07 |  | 0.23 | ± | 0.07 |  | 0.24 | ± | 0.07 | 0.23 | ± | 0.09 |  | 0.26 | ± | 0.07 |  | 0.24 | | ± | 0.07 | 0.24 | | ± | 0.08 |  | 0.25 | | ± | 0.07 |  |
| Ln-HF (ms^2^) | 5.93 | ± | 0.99 | 5.07 | ± | 1.42 | * | 6.05 | ± | 1.25 |  | 6.13 | ± | 1.02 | 5.33 | ± | 1.09 | * | 5.66 | ± | 1.25 |  | 6.49 | | ± | 1.06 | 4.72 | | ± | 0.84 | * | 5.81 | | ± | 1.05 | * |
| Ln-LF (ms^2^) | 6.72 | ± | 1.02 | 6.51 | ± | 1.06 |  | 7.41 | ± | 0.89 |  | 6.84 | ± | 1.02 | 6.92 | ± | 0.96 |  | 7.15 | ± | 1.03 |  | 7.18 | | ± | 0.91 | 6.65 | | ± | 0.88 |  | 7.13 | | ± | 0.93 |  |
| Ln-LF/HF | 1.21 | ± | 0.42 | 1.80 | ± | 0.93 | * | 1.64 | ± | 0.65 | * | 1.18 | ± | 0.47 | 1.84 | ± | 0.79 | * | 1.74 | ± | 0.64 | * | 1.17 | | ± | 0.69 | 2.10 | | ± | 0.51 | * | 1.62 | | ± | 0.64 | * |
| Ln-TP (ms^2^) | 7.91 | ± | 0.85 | 7.52 | ± | 0.90 |  | 8.45 | ± | 0.89 |  | 7.94 | ± | 0.95 | 8.01 | ± | 1.29 |  | 8.18 | ± | 1.02 |  | 8.14 | | ± | 0.84 | 7.24 | | ± | 0.67 | * | 8.32 | | ± | 0.80 |  |
| Values are Mean ± SD. *: ≠ PRE; #: ≠ N; $≠ H-HR; p<0.05; RR: R-R interval; SDNN: standard deviation of normal to normal R-R intervals; RMSSD: square root of the mean squared differences of successive NN intervals; Ln: natural logarithm transformation;; HF: High-frequency spectral power; LF: Low-frequency spectral power; TP: Total spectral power. | | | | | | | | | | | | | | | | | | | | | | | | | | | | | | | | | | | | |
|  |  |  |  |  |  |  |  |  |  |  |  |  |  |  |  |  |  |  |  |  |  |  |  |  |  |  |  |  |  |  |  |  |  |  |  |  |
|  |  |  |  |  |  |  |  |  |  |  |  |  |  |  |  |  |  |  |  |  |  |  |  |  |  |  |  |  |  |  |  |  |  |  |  |  |
